# Mediation of lubricated air films using spatially periodic dielectrophoretic effect

**DOI:** 10.1038/s41467-021-24534-6

**Published:** 2021-07-13

**Authors:** Quoc Vo, Tuan Tran

**Affiliations:** 1 School of Mechanical & Aerospace Engineering, HP-NTU Digital Manufacturing Corporate Lab, Nanyang Technological University, Singapore, Singapore; 2 Division of Physics and Applied Physics, School of Physical and Mathematical Sciences, Nanyang Technological University, Singapore, Singapore

**Keywords:** Applied physics, Surfaces, interfaces and thin films, Fluid dynamics

## Abstract

A stone thrown in a lake captures air as it collides with water and sinks; likewise a rain drop falling on a flat surface traps air bubbles underneath and creates a spectacular splash. These natural occurrences, from bubble entrapment to liquid ejection, happen as air fails to escape from the closing gap between liquid and solid surfaces. Trapping of air is devastating for casting, coating, painting, and printing industries, or those intolerant of water entry noise. Attempts to eliminate the interfering air rely on either reducing the ambient pressure or modifying the solid surfaces. The former approach is inflexible in its implementation, while the latter one is inherently limited by the wetting speed of liquid or the draining capacity of air passages created on the solid. Here, we present a “divide and conquer” approach to split the thin air gap into tunnels and subsequently squeeze air out from the tunnels against its viscous resistance using spatially periodic dielectrophoretic force. We confirm the working principles by demonstrating suppression of both bubble entrapment and splash upon impacts of droplets on solid surfaces.

## Abstract (web summary)

The violent splash of a droplet caused by residual air pockets trapped during impact on a solid surface appears inevitable. Vo and Tran show how to vent the drop on short notice for a smooth touchdown, harnessing dielectrophoretic forces to create dynamic drainage channels.

## Introduction

In 2012, the unexpected discovery of thousands of micro-cracks in high-pressure boilers of Belgium’s two nuclear reactors brought their operations to a complete halt for fear of catastrophic nuclear accidents and bewildered both the public and the scientific community^1,2^. Although inconclusive, numerous investigations attributed gas pockets in the carbon steel shells as the initiating cause of the cracks^3,4^. Gas pockets are commonly formed in the early stage of metal production in which the process of pouring molten metal entrains and traps the surrounding gas in the metal^5^. Metal production then joins a long list of industries, e.g. coating, painting and printing^5–8^, falling victims to a seemingly insignificant but persistent problem of gas inclusion in the final products.

Typically, when a deformable surface moves against another, either deformable or rigid, gas is squeezed out from the gap between the two surfaces until viscous resistance dominates and hinders the gas flow^6^. This phenomenon is so robust that it happens either for normal impact or in the case that one surface moves along the other. Notorious examples of the former scenario include bubble entrapment^9–11^ and bouncing of impacting droplets^12^, whereas those exemplifying the latter are deflection of spreading lamella in droplet splashing^13,14^ and entrapment of air film when solid surfaces plunging into liquid^8,15^. Attempts to eliminate the interfering air rely on either reducing the ambient pressure^16,17^ or modifying the solid surfaces to help squeezing air out of the closing gap. The latter approach, with greater flexibility in its implementation, has been explored by enhancing the solid’s wettability^18^, changing surface stiffness^19^, or fabricating escaping air passages on the solid surfaces^20^. This approach, however, is inherently limited by the wetting speed of liquid or the draining capacity of the fabricated passages.

To prevent adverse effects of air entrapment, we use the dielectrophoretic effect to deform the liquid surface when it is sufficiently close to the solid surface, effectively inducing controlled ruptures to the air film and creating escaping tunnels for air. We demonstrate, experimentally and theoretically, that the proposed method effectively minimises both bubble entrapment and splash formation, the most common phenomena caused by air failing to escape from the gap between liquid and solid surfaces.

## Results and discussion

### Air film mediation by the dielectrophoretic effect

**Fig. 1:**
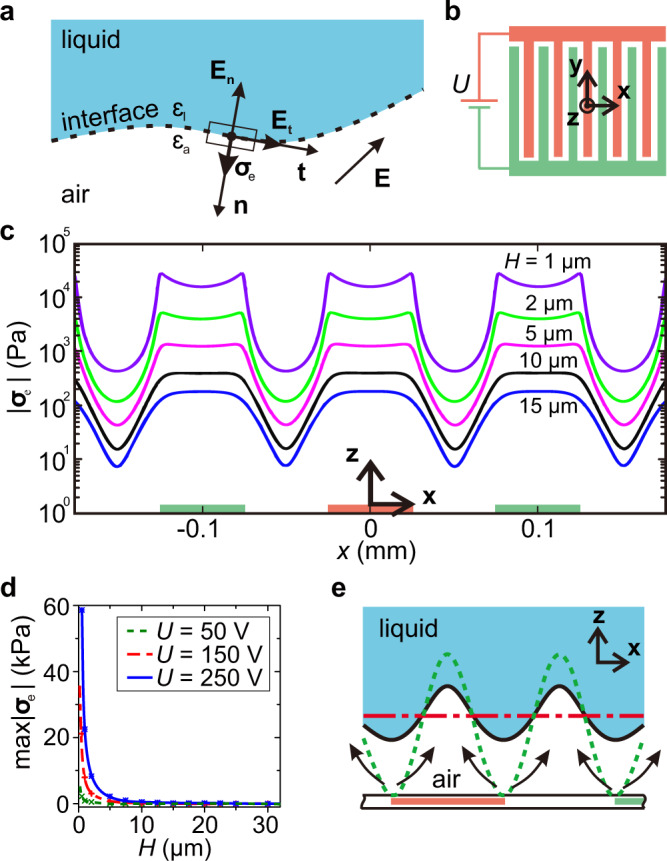
Spatially periodic dielectrophoretic stress generated by an interdigital electrode substrate. **a** Schematic of the stress ***σ***_e_ caused by an electric field **E** at the interface of two media, liquid and air, with different dielectric constants. **b** Schematic of electrodes fabricated on a substrate to create a spatially periodic electric field. **c** Simulated dielectrophoretic stress magnitude ∣***σ***_e_∣ vs. *x* for a liquid–air interface at different distances *H* from the substrate and *U* = 200 V. For *H* ⪅ 10 μm, ∣***σ***_e_∣ peaks directly above the electrodes’ edges. **d** Simulated maximum dielectrophoretic stress magnitude $\max (| {{\boldsymbol{\sigma }}}_{{\rm{e}}}| )$ vs. *H* for several values of *U*. **e** A thin air film between a liquid surface and a solid surface ruptures where the dielectrophoretic stress eventually becomes strongest, i.e., directly above the electrodes’ edges.

At the interface of two media with different dielectric constants, activating an electric field **E** generates a dielectrophoretic stress on the interface within several pico-seconds^21^. The induced dielectrophoretic stress, denoted as ***σ***_e_, is perpendicular to the interface and points towards the medium with lower dielectric constant^22^. In particular, for a liquid–air interface, the dielectrophoretic stress is calculated using Maxwell stress tensor (see Supplementary Note 1 for details)^22–26^:

1 $${{\boldsymbol{\sigma }}}_{{\rm{e}}}=\frac{1}{2}{\varepsilon }_{0}\left[{E}_{{\rm{l,t}}}^{2}({\varepsilon }_{{\rm{l}}}-{\varepsilon }_{{\rm{a}}})+{E}_{{\rm{l,n}}}^{2}{\varepsilon }_{{\rm{l}}}\left(\frac{{\varepsilon }_{{\rm{l}}}}{{\varepsilon }_{{\rm{a}}}}-1\right)\right]\ \widehat{{\bf{n}}},$$

where *E*_l,t_ and *E*_l,n_, respectively, are the tangential and normal components of the electric field **E**_l_ inside the liquid at the interface, *ε*_0_ is the permittivity of vacuum, *ε*_a_ and *ε*_l_, respectively, are the dielectric constants of air and the liquid, and $\widehat{{\bf{n}}}$ denotes the interface’s normal vector (Fig. 1a). In principle, the mechanism generating the dielectrophoretic stress suggests that a sufficiently strong and spatially varying electric field guarding a solid surface may deform an adjacent liquid surface, thereby mediating the intervening air layer between the two surfaces.

To create a spatially periodic electric field on a solid surface, we use a pair of interdigital electrodes fabricated on the surface (Fig. 1b and “Methods”). The width and the interspacing of electrodes, equal and denoted as *δ*, are sufficiently small (*δ* = 50 μm in our experiment) to create a strong dielectrophoretic stress when a direct current (DC) voltage is applied across the pair of electrodes. For example, for a water–air interface at a distance 2 μm from the solid substrate, applying 200 V to the electrodes yields the peak value of dielectrophoretic stress ≈10 kPa. Here we note that the electric field exponentially decays into the space above the substrate with an effective penetration depth *d*_p_ = 2*δ*/*π* ≈ 32 μm^25^. Since *ε*_a_ < *ε*_l_, Eq. (1) also indicates that the dielectrophoretic stress on such liquid–air interface always points towards the solid surface; its magnitude ∣***σ***_e_∣ periodically varies in the *x*− direction (see Fig. 1b for the coordinates) and depends on both the applied voltage *U* and the distance *H* to the substrate. For a fixed value of *H* satisfying *H* ⪅ 10 μm, ∣***σ***_e_∣ peaks above the electrodes’ edges and plunges to its minima at the centres of the electrodes and their gaps (Fig. 1c). Notably, the maximum magnitude of the dielectrophoretic stress $\max (| {{\boldsymbol{\sigma }}}_{{\rm{e}}}| )$ increases exponentially as *H* → 0 (Fig. 1d).

The dielectrophoretic stress’s periodic variation is the key to induce controlled rupture of an air film and split it into smaller tunnels (Fig. 1e). An otherwise uniform dielectrophoretic stress over the liquid surface is incapable of collapsing the air film on a perfectly smooth solid surface because it has a finite maximum value at *H* = 0, whereas the viscous resistance in the air diverges with *H*^−3^ as *H* → 0 (see “Methods”). Likewise, an electrostatic stress, which is generated by oppositely charging the liquid and the surface^27^, diverges with *H*^−2^ and is therefore incapable of overcoming the air’s viscous resistance for an air film of uniform thickness.

We highlight that the dielectrophoretic effect in principle works for any type of liquid, as long as there is a discontinuity in dielectric constant at the liquid–air interface. This is directly inferred from Eq. (1) and experimentally verified using a wide range of liquids, including both conductive and non-conductive types (see Supplementary Fig. 2 for details).

### Elimination of bubble entrapment

**Fig. 2:**
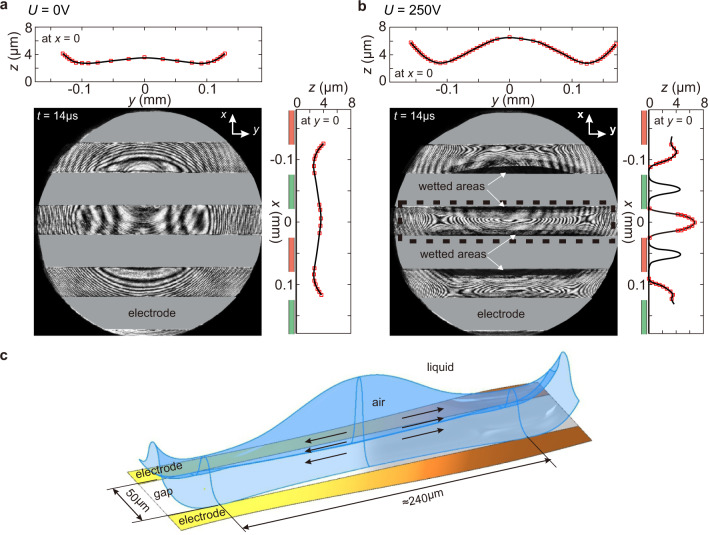
Spatially periodic dielectrophoretic effect on air films under impacting droplets. **a** Without dielectrophoretic effect (*U* = 0): interferometric pattern and extracted profiles of an air film formed between an impacting ethanol droplet and an interdigital electrode substrate. There is no wetting at this moment and the air film profile appears axisymmetrical. **b** With dielectrophoretic effect (*U* = 250 V): interferometric pattern and extracted air profiles show that wetting already occurs along the electrode’s edges (indicated by arrows). The air layer splits into tunnels on top of the electrodes and the gaps. In both **a**, **b**, *t* = 14 μs, *R* = 1.3 mm, *v* = 1.4 m s^−1^; optical access to the air film is partially blocked by the electrodes (the grey areas). **c** Three-dimensional profile of the central air tunnel reconstructed from the interferogram. The air tunnel occupies the area indicated by the dashed box in **b**.

We now demonstrate the dielectrophoretic effect on the thinning air gap between a solid surface and a liquid droplet of initially negligible potential impacting the surface in the normal direction. We compare the bottom-view snapshots of impacts with and without the dielectrophoretic effect; both snapshots were taken after a fixed duration (14 μs) from the time origin, set at the moment the droplet’s lower surface was first detected by interferometric signals (see “Methods”). Without the dielectrophoretic effect (*U* = 0 V), the air trapped between the liquid and the solid surface resembles a round dimple (Fig. 2a). Subsequent rupture of the air film occurs at the dimple’s neck where the film thickness is smallest, an observation consistent with previous studies on bubble entrapment^9,28,29^ (see Supplementary Movie 1). In contrast, the snapshot taken with the dielectrophoretic effect (*U* = 250 V) presents a strikingly distinct picture. Wetting already occurs along the electrodes’ edges (Fig. 2b), effectively creating air tunnels along the electrodes. A representative three-dimensional profile of the central air tunnel is reconstructed from the captured interference pattern and shown in Fig. 2c. Observation of the resulting air tunnels thus confirms our prediction of the dielectrophoretic effect on the liquid surface shown in Fig. 1e: it is strongest above the electrodes’ edges and capable of accelerating the liquid against not only the air flow’s viscous resistance but also the Laplace pressure generated with the deformed liquid surface.

**Fig. 3:**
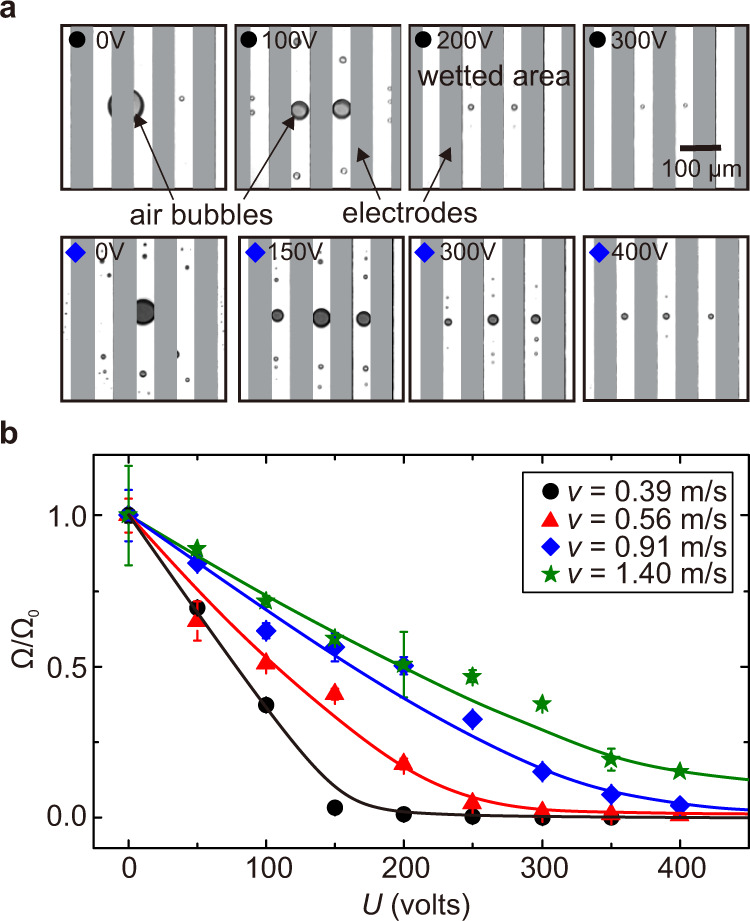
Elimination of bubble entrapment using the periodic dielectrophoretic effect. **a** Bottom-view snapshots taken 15 ms after impacts of *R* = 1.3 mm ethanol droplets for several values of *U* and for two values of impact velocities, i.e., *v* = 0.39 m s^−1^ (upper panel) and *v* = 0.91 m s^−1^ (lower panel). The surfaces in these snapshots are already wetted except the areas occupied by air bubbles. At *U* = 0 V, the trapped air film contracts to a large air bubble with radius ≈45 μm; increasing *U* to 400 V significantly reduces the bubble’s radius. **b** Plot showing the volume ratio Ω/Ω_0_ of the trapped bubbles vs. *U* for several values of *v*. Here Ω_0_ and Ω are total volumes of air trapped at *U* = 0 and *U* > 0, respectively. The error bars represent the standard deviations. The solid lines are to guide the eye. All the impacts shown here do not exhibit bouncing behaviour.

The rupture of air film into small tunnels facilitates draining of air and effectively reduces the final volume of air entrapped under the liquid. In Fig. 3a, we show two series of snapshots of the areas impacted by falling droplets with two different velocities, *v* = 0.39 m s^−1^ (upper panel) and *v* = 0.91 m s^−1^ (lower panel), for several values of applied voltage. The snapshots had been all taken at *t* ≈ 15 ms after wetting was first initiated; at this moment, air entrapment had already been completed, resulting in bubbles on the surface. At *U* = 0 V and *v* = 0.39 m s^−1^, the trapped air film contracts to form a large bubble of radius ≈45 μm, whereas activating the dielectrophoretic effect splits the air film and forms significantly smaller bubbles along the electrode gaps. We also observe that an impact of greater velocity needs higher voltage to achieve similar effect, e.g. for *v* = 0.39 m s^−1^, increasing *U* from 0 to 100 V causes an ≈27-fold reduction in the total volume of trapped air (from 340 to 12.6 pL), whereas, for *v* = 0.91 m s^−1^, 400 V is required to achieve a similar ratio of volume reduction (from 160 to 6.5 pL). In Fig. 3b, we show that increasing *U* consistently reduces the total volume of trapped air for several values of impact velocity. This test confirms that the dielectrophoretic effect can be used to practically eliminate air entrapment between surfaces directly approaching each other.

### Splash suppression

**Fig. 4:**
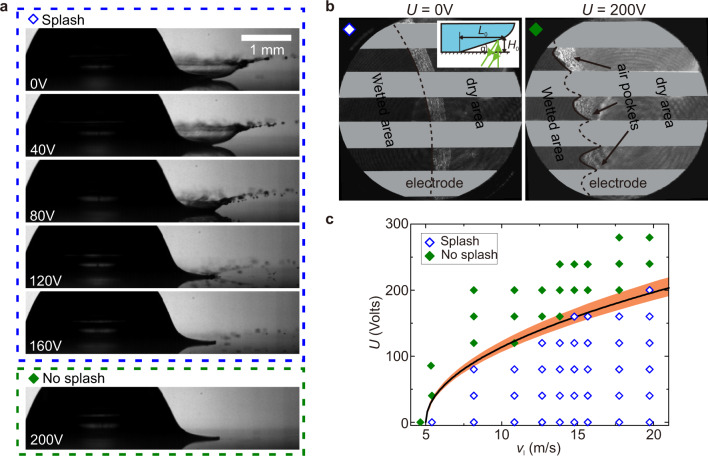
Splash suppression using the periodic dielectrophoretic effect. **a** Snapshots showing progressive suppression of splash by increasing *U* from 0 to 160 V. Splash is completely eliminated at *U* = 200 V. The impacting droplets have *R* = 1.3 mm and *v* = 3.1 m s^−1^; the snapshots are all taken at *t* = 0.3 ms after impact. **b** Bottom-view snapshots taken at *t* = 30 μs after impacts of droplets with *v* = 2.5 m s^−1^ for *U* = 0 V (left snapshot) and *U* = 200 V (right snapshot). Applying the voltage causes significant changes of the wetting front and the resulting air layer between the advancing lamella and the surface. In both snapshots, the wetting radius is ≈500 μm. Inset: Schematic of the air wedge made by the spreading lamella and the substrate. The small wedge angle *α* allows formation of interference patterns in front of spreading lamellas. **c** Diagram showing a clear separation between splash and no-splash behaviours when varying the spreading velocity *v*_l_ of the lamella and the applied voltage *U*. The solid line indicates the splash suppression condition ${U}_{{\rm{m}}}={C}^{-1/2}{{\Phi }}{({v}_{{\rm{l}}}-{v}_{{\rm{t}}})}^{1/2}$, where *C* = 1.51. The shaded area indicates ±10% deviation from the theoretically predicted transition.

The dielectrophoretic force effectively eliminates air entrapment and its adverse consequences even for a liquid surface moving along a solid one. In this case, the relative motion between the liquid and the solid surfaces causes a lubrication pressure build-up in the wedge formed by the liquid front and the solid. Air entrapment or deflection of the liquid is ensured for sufficiently high relative velocity. The formation of splash upon impact of a droplet on a solid surface is a notorious manifestation of the pressure build-up and subsequent deflection of the fast spreading liquid lamella^16,17,30^. Nonetheless, activating the dielectrophoretic effect by increasing the applied voltage *U* from 0 to 200 V gradually diminishes and eventually suppresses lamella ejection, causing the impacting droplet to spread smoothly (Fig. 4a and Supplementary Movie 2). A close look at the air layer that precedes the expanding lamella using interferometric recordings reveals a drastic change in the wetting front caused by the dielectrophoretic effect: from a circular ring (*U* = 0 V) to a saw-tooth shape (*U* = 200 V) with each tooth pointing to an electrode edge (Fig. 4b). The observed change indicates that the suppression of splash directly connects to ruptures of the air film along the electrode edges and the resulting air flow dynamics.

There are two key factors in suppressing deflection of the expanding lamella: (1) the induced dielectrophoretic force pulls the advancing lamella down against a combination of aerodynamical lift on top of the expanding lamella and lubrication force in the air underneath^30^ and (2) the spatial variation of the dielectrophoretic force enables it to overcome the lubrication force as the air film thickness diminishes, forcing the liquid to wet the surface along the electrode edges. The condition to suppress splashes, therefore, is written as *F*_d_ ≥ *F*_l_, where *F*_d_ is the dielectrophoretic force pulling the liquid down and *F*_l_ the excess lubrication force pushing the lamella upward. Our calculation (see “Methods”) shows that *F*_d_ acting on the liquid lamella above an air wedge of small angle *α* and height *H*_0_ (see inset of Fig. 4b) depends quadratically on the applied voltage *U* as

2 $${F}_{{\rm{d}}}=C{K}_{{\rm{e}}}{\varepsilon }_{0}{\varepsilon }_{{\rm{l}}}({\varepsilon }_{{\rm{l}}}-1)\frac{{U}^{2}}{{d}_{{\rm{p}}}},$$

where *C* is a constant of order unity resulting from approximating the electric potential in the liquid by the first-order Fourier expansion^25^, *K*_e_ is a dimensionless term that depends on the involving material properties, the penetration depth *d*_p_ and the air wedge’s geometry (see “Methods”). The excess lubrication force *F*_l_ linearly depends on the expanding velocity *v*_l_ of the lamella^30^:

3 $${F}_{{\rm{l}}}={K}_{{\rm{l}}}\ {\eta }_{{\rm{a}}}({v}_{{\rm{l}}}-{v}_{{\rm{t}}}),$$

where ${K}_{{\rm{l}}}=6\ {\mathrm{ln}}\,(1+{H}_{0}/4.8{\lambda }_{{\rm{a}}})/{\tan }^{2}\alpha$, *λ*_a_ = 68 nm is the mean free path of air at atmospheric pressure and room temperature, *η*_a_ is the viscosity of air and *v*_t_ the threshold velocity of the lamella above which splash occurs without the dielectrophoretic force^30^. In our study, the threshold velocity *v*_t_ = 5.0 ± 0.18 m s^−1^ and the angle *α* = 5.7 ± 0. 6° are determined experimentally (see “Methods”). The condition *F*_d_ ≥ *F*_l_ therefore translates to one for the minimum applied voltage *U*_m_ that causes splash suppression:

4 $${U}_{{\rm{m}}}={C}^{-1/2}{{\Phi }}{({v}_{{\rm{l}}}-{v}_{{\rm{t}}})}^{1/2}.$$

The term ${{\Phi }}={[{K}_{{\rm{l}}}{\eta }_{{\rm{a}}}{d}_{{\rm{p}}}/{K}_{{\rm{e}}}{\varepsilon }_{0}{\varepsilon }_{{\rm{l}}}({\varepsilon }_{{\rm{l}}}-1)]}^{1/2}$, determined entirely by the system parameters, reflects how the splash suppression condition depends on the material properties of the involving phases and geometrical features of the spreading lamella.

In Fig. 4c, we show a diagram highlighting separation between the splash and no-splash behaviours of ethanol droplets impacting on an interdigital electrode substrate. The recorded behaviours are obtained by varying the applied voltage *U* from 0 to 280 V and lamella velocity *v*_l_ from 4.63 to 19.75 m s^−1^. The solid line indicates the minimum voltage *U*_m_ determined by Eq. (4). The predicted condition is remarkably consistent with the experimentally observed transition from splash to no splash, confirming the working principle of periodic dielectrophoretic force in rupturing the air film and preventing the lamella’s deflection.

We have demonstrated a “divide and conquer” method to mediate the air film between two approaching surfaces. By using a periodic dielectrophoretic force, we effectively induce spatially controlled ruptures of the air film and suppress adverse outcomes of air interference, e.g. bubble entrapment and splash formation, regardless of the surfaces’ approaching direction. The proposed method, empowered by versatile electrode designs and flexibility in working liquids, is in principle capable of imposing any rupturing pattern to air films, thus have strong bearing on control of air inclusion or manipulations of liquid–surface interactions.

## Methods

### Experimental

The substrate is a glass wafer covered with an interdigital array of gold electrodes of thickness *d*_c_ = 250 nm. The electrode width and interspacing are *δ* = 50 μm (Fig. 1b). An insulating layer of silicon nitride (Si_3_N_4_) of thickness 500 nm is deposited on top of the substrate and electrodes using plasma-enhanced chemical vapour deposition. The silicon nitride layer is covered by a 50-nm layer of fluoropolymer (Teflon, Dupont). The dielectrophoretic effect is induced by applying a DC voltage varying from 0 to 400 V to the electrode terminals, which are connected to the pair of interdigital electrodes. The needle used to dispense droplets is grounded to ensure negligible initial potential for impacting droplets.

#### High-speed imaging experiment

The interactions between impacting droplets and the solid surface are observed from the bottom view using two interferometric set-ups: set-up 1 is used to capture snapshots with high spatial resolutions, both in horizontal and vertical directions, and set-up 2 is used to track the fast evolution of the air layer profiles.

In set-up 1, we use a high-speed camera (SAX2, Photron Inc.) illuminated by a dual-pulse laser (Nd:YLF DualPower TR, Dantec Dynamics Inc.) with 4 ns pulse width. In this set-up, the coherence length of the laser is ≈10 mm, sufficiently large to enable capture of the entire interference patterns of the air tunnels, which can be as thick as 10 μm, as shown in Fig. 2a, b.

In set-up 2, we use an ultra-high-speed camera (Kirana, Specialised Imaging) running at the frame rate of 2 million frames per second (FPS) and image size of 924 px × 768 px. The camera is synchronised with an ultra-high-speed laser diode (Cavilux Smart UHS, Cavitar Inc.) with 10 ns pulse width. The inter-frame time of this set-up (500 ns) is sufficiently small, allowing us to follow minute changes of the recorded interference patterns between frames and subsequently determine the moment wetting is initiated. The air film profile is constructed at the moment wetting is first initiated. Time-dependent film profiles in absolute unit are then progressively constructed backwards in time^9^. By matching the size and shape of these time-dependent film profiles to those obtained by set-up 1 for impacts of identical conditions, the pre-wetting film profile shown in Fig. 2a is obtained.

We used two cameras (SA5 and SAX2, Photron Inc.) running at 100,000 FPS to capture synchronous side-view recordings of the impact behaviours in two directions: one along and the other one perpendicular to the electrodes^31^. We observed that for the same impact conditions, the required voltage to suppress liquid ejection along the electrodes is slightly higher than that perpendicular to the electrodes. In other words, the condition to suppress ejection of liquid moving along the electrodes, i.e. along the *y*– axis, can be sufficiently used as the overall condition to suppress splash.

#### Determination of time origin

For an approaching droplet, its lower surface is first detected by set-up 2 when interferometric signals created by reflected light from the solid–air and air–liquid interfaces are visible in the recorded images; this moment is reliably taken as *t* = 0 because the fringes are only possible when the liquid–solid distance becomes <20 μm, i.e. half the coherence length of the illuminating laser (Cavilux Smart UHS, Cavitar Inc.).

By defining *t* = 0 the moment the fringes first appear (≈20 μm above the solid surface), we are able to have a reference point independent of the dielectrophoretic effect. The results shown using this time origin help to highlight the acceleration of the liquid–air interface and the dielectrophoretic force that causes such an acceleration. For example, given impacts of the same velocity and drop size, Fig. 2a shows that, after 14 μs, the liquid–air interface has not touched the solid without the dielectrophoretic force (*U* = 0 V), whereas Fig. 2b shows that it already touches the solid when the dielectrophoretic force is activated (*U* = 250 V).

As a result, we use this approach to define the time reference when comparing impact behaviours with varying voltage.

### Numerical simulations of electric field and dielectrophoretic stress

The electric potential ϕ and electric field **E** generated by the interdigital electrodes and the resulting dielectrophoretic stress on the liquid–air interface are simulated using module DC/alternating current of COMSOL 4.3b. The simulation model uses the same dimensions and material properties of the system, including those of the electrodes, coating and substrate materials. The working liquid is ethanol having dielectric constant *ε*_l_ = 24.5. The liquid–air interface is parallel with the substrate. The distance *H* from the liquid–air interface to the substrate is varied from 0.5 to 32 μm. Typical simulated results of ϕ and **E** are shown in Supplementary Fig. 1. The magnitude of the dielectrophoretic stress ∣***σ***_e_∣ shown in Fig. 1 is calculated using Eq. (1) and the simulated electric field **E**.

### Derivation of the dielectrophoretic stress

For a liquid–air interface parallel with a solid substrate, a simplified expression for the dielectrophoretic stress is obtained by approximating the electric field in the liquid by its first-order Fourier term and substituting it to Eq. (1) (see Supplementary Note 1):

5 $${{\boldsymbol{\sigma }}}_{{\rm{e}}}\approx {\varepsilon }_{0}{\left(\frac{{\varepsilon }_{{\rm{r}}}}{{\varepsilon }_{{\rm{l}}}}\right)}^{2}({\varepsilon }_{{\rm{a}}}-{\varepsilon }_{{\rm{l}}})\frac{16{U}^{2}}{{\pi }^{4}{d}_{{\rm{p}}}^{2}}\exp \left(-\frac{2H}{{d}_{{\rm{p}}}}\right)\left[{\sin }^{2}\left(\frac{x}{{d}_{{\rm{p}}}}\right)+\frac{{\varepsilon }_{{\rm{l}}}}{{\varepsilon }_{{\rm{a}}}}{\cos }^{2}\left(\frac{x}{{d}_{{\rm{p}}}}\right)\right]\ \widehat{{\bf{z}}},$$

where *ε*_r_ = 2*ε*_a_*ε*_l_/[*ε*_a_(*b* + 1) + *ε*_l_(1 − *b*)] with $b=\exp (-2H/{d}_{{\rm{p}}})$; $\widehat{{\bf{z}}}=-\widehat{{\bf{n}}}$ is the unit vector in the *z*− direction. Here ***σ***_e_ has the same sign with *ε*_a_ − *ε*_l_, indicating that the dielectrophoretic stress is directed towards the medium having lower dielectric constant, i.e. from liquid to air. This is consistent with the experimental observation by Brown et al.^22^. In our case, this implies that ***σ***_e_ always points downward to the solid substrate. The maximum dielectrophoretic stress resulting from Eq. (5) is

6 $$\max (| {{\boldsymbol{\sigma }}}_{{\rm{e}}}| )={\varepsilon }_{0}\frac{{\varepsilon }_{{\rm{r}}}^{2}}{{\varepsilon }_{{\rm{l}}}}\left(\frac{{\varepsilon }_{{\rm{l}}}}{{\varepsilon }_{{\rm{a}}}}-1\right)\frac{16{U}^{2}}{{\pi }^{4}{d}_{{\rm{p}}}^{2}}\exp \left(-\frac{2H}{{d}_{{\rm{p}}}}\right),$$

which is achieved at the electrode centres, i.e., *x* = *mπd*_p_ = 2*mδ*, where *m* = 0, ±1, ±2, ....

It is important to note that, at a small distance to the surface (*H* ⪅ 10 μm), a higher-order expansion of the electric field is required to obtain a more accurate approximation for the maximum dielectrophoretic stress^25^. In particular, the peak values of the dielectrophoretic stress move from the centres to the edges of the electrodes when *H* reduces and becomes <10 μm (see Fig. 1c). This is the key reason leading to ruptures of the air film at the electrode edges instead of the electrode centres and is consistent with previously reported experimental observations^22^. As the expression for the stress using higher orders of the Fourier expansion is analytically intractable, we take a simplified approach by using the first-order Fourier expansion (Eq. (6)) and introducing a factor *C* to account for the absence of higher-order contributions. In other words, the dielectrophoretic stress effectively responsible for ruptures of the thin air film at the electrode edges is

7 $${\sigma }_{{\rm{d}}}\approx C{\varepsilon }_{0}\frac{{\varepsilon }_{{\rm{r}}}^{2}}{{\varepsilon }_{{\rm{l}}}}\left(\frac{{\varepsilon }_{{\rm{l}}}}{{\varepsilon }_{{\rm{a}}}}-1\right)\frac{16{U}^{2}}{{\pi }^{4}{d}_{{\rm{p}}}^{2}}\exp \left(-\frac{2H}{{d}_{{\rm{p}}}}\right).$$

### Comparison between lubrication pressure and dielectrophoretic stress

Consider a thin air film of uniform thickness sandwiched between a solid substrate and a liquid surface approaching with velocity *v;* the lateral velocity of air flows in the film is ~2*Lv*/*H*, where *H* and *L* are the film’s thickness and length, respectively. The resulting lubrication pressure in the air film is *p* ~ *η*_a_*L*^2^*v*/*H*^3^, where *η*_a_ is the dynamic viscosity of air.

Now we assume that an *uniform* dielectrophoretic stress *σ*_d_ is applied on the liquid–air interface. As the thickness of the air film between the liquid and the solid diminishes, the dielectrophoretic stress is capped at $C{\varepsilon }_{0}{\varepsilon }_{{\rm{l}}}({\varepsilon }_{{\rm{l}}}-1){(4U/{\pi }^{2}{d}_{{\rm{p}}})}^{2}$ (see Eq. (7)), whereas the lubrication pressure in the film grows with *H*^−3^. Therefore, the dielectrophoretic stress eventually becomes smaller than the lubrication pressure at a sufficiently small thickness.

In contrast, with the periodic dielectrophoretic stress induced by interdigital electrodes, the liquid surface directly above the electrode edges first bulges towards the solid surface, forcing air flow laterally from the space above the electrode edges to that above the centres of the electrodes and their gaps (see Fig. 1e). The main resistive factor against surface deformation caused by the periodic dielectrophoretic stress is the Laplace pressure. Nonetheless, the Laplace pressure is insignificant compared to the dielectrophoretic stress. For instance, for an ethanol surface at *H* = 2 μm, the dielectrophoretic stress *σ*_d_ caused by applying *U* = 200 V is 5114 Pa, an order of magnitude larger than the Laplace pressure (≈140 Pa) assuming maximum curvature for surface deformation (see Supplementary Note 2). As a result, the periodic dielectrophoretic stress inevitably ruptures the air film at the electrode edges and forms air tunnels along the electrodes and their gaps.

### Dielectrophoretic force acting on a lamella

We consider a lamella advancing on a solid substrate with velocity *v*_l_ at the moment it starts deflecting upwards. The lower surface of the lamella makes an air wedge of a small angle *α* and height *H*_0_ with the surface (see Fig. 4b, inset). The dielectrophoretic force per unit length at the electrode edges pulling the lamella downwards is calculated by integrating the dielectrophoretic stress *σ*_d_ (Eq. (7)) along the lamella’s lower surface:

8 $${F}_{{\rm{d}}}=\mathop{\int }\nolimits_{{\rm{0}}}^{{L}_{0}}{\sigma }_{{\rm{d}}}\ {\rm{d}}L\approx C\frac{1}{\tan \alpha }\frac{{\varepsilon }_{0}}{{\varepsilon }_{{\rm{l}}}}\left(\frac{{\varepsilon }_{{\rm{l}}}}{{\varepsilon }_{{\rm{a}}}}-1\right)\frac{16{U}^{2}}{{\pi }^{4}{d}_{{\rm{p}}}^{2}}\mathop{\int }\nolimits_{{\rm{0}}}^{{H}_{0}}{\varepsilon }_{{\rm{r}}}^{2}\exp \left(-\frac{2H}{{d}_{{\rm{p}}}}\right){\rm{d}}H.$$

Here ${L}_{0}={H}_{0}/\tan \alpha$. As a result, we obtain

9 $${F}_{{\rm{d}}}\approx C{K}_{{\rm{e}}}{\varepsilon }_{0}{\varepsilon }_{{\rm{l}}}({\varepsilon }_{{\rm{l}}}-1)\frac{{U}^{2}}{{d}_{{\rm{p}}}},$$

where

10 $${K}_{{\rm{e}}}=\frac{16}{{\pi }^{4}}\frac{1}{\tan \alpha }\ \frac{1-\exp \,(-2{H}_{0}/{d}_{{\rm{p}}})}{{\varepsilon }_{{\rm{a}}}+{\varepsilon }_{{\rm{l}}}+({\varepsilon }_{{\rm{a}}}-{\varepsilon }_{{\rm{l}}})\exp \,(-2{H}_{0}/{d}_{{\rm{p}}})}.$$

### Excess lubrication force acting on a lamella

The lubrication force inside the air wedge pushing the lamella upward is calculated using the lubrication approximation for the velocity field in the wedge and the slip boundary condition at the solid surface^30^:

11 $${F}_{{\rm{lub}}}={K}_{{\rm{l}}}\ \ {\eta }_{{\rm{a}}}{v}_{{\rm{l}}},$$

where

12 $${K}_{{\rm{l}}}=\frac{6}{{\tan }^{2}\alpha }{\mathrm{ln}}\,\left(1+\frac{{H}_{0}}{4.8{\lambda }_{{\rm{a}}}}\right).$$

Here *λ*_a_ = 68 nm is the mean free path of air at atmospheric pressure and room temperature and *v*_l_ is the lamella’s spreading velocity. Without the dielectrophoretic effect, splash occurs when the lubrication force overcomes the capillary force keeping the lamella from deflecting upward^30^. One can determine the maximum capillary force preventing splash by considering the splash threshold:

13 $${F}_{{\rm{cap}}}={K}_{{\rm{l}}}\ {\eta }_{{\rm{a}}}{v}_{{\rm{t}}},$$

where *v*_t_ is the lamella velocity at the splash threshold, i.e., splash occurs when *v*_l_ > *v*_t_.

To suppress splash, an activated dielectrophoretic force has to overcome the excess lubrication force causing lamella deflection:

14 $${F}_{{\rm{l}}}={F}_{{\rm{lub}}}-{F}_{{\rm{cap}}}={K}_{{\rm{l}}}\ {\eta }_{{\rm{a}}}({v}_{{\rm{l}}}-{v}_{{\rm{t}}}).$$

### Measurement of wedge angle *α* and the wedge’s height *H*_0_

The wedge angle *α* under an expanding lamella is measured by high-speed interferometric imaging. In Supplementary Fig. 3a, we show an interferometric bottom-view snapshot under the spreading lamella of a droplet splashing on a teflon-coated glass substrate. The image was taken the moment the lamella starts ejecting upward from the substrate (≈30 μs after the liquid touches the solid). In this experiment, the impact velocity is *v* = 3.1 m s^−1^, and the spreading velocity of the lamella is *v*_l_ = 13 m s^−1^. In Supplementary Fig. 3b, we show the averaged air layer’s profile extracted from the captured interference pattern. The measured air wedge’s angle is *α* = 5.7 ± 0. 6° and wedge’s height is *H*_0_ = 8.5 μm.

## Supplementary information

Supplementary Information

Supplementary Movie 1

Supplementary Movie 2

Description of Additional Supplementary Files

## Data Availability

All processed data in this study are included in this published article (and its supplementary information files). Raw data will be provided upon reasonable requests.
