# Supplementary material for: Mediation of lubricated air films using spatially periodic dielectrophoretic effect
Source: Nat Commun. 2021 Jul 13;12:4289. doi: 10.1038/s41467-021-24534-6 (PMC8277893; doi:10.1038/s41467-021-24534-6)
Supplement: Supplementary file 1 — Supplementary Information [file 41467_2021_24534_MOESM1_ESM.pdf]

Mediation of lubricated air films using spatially periodic dielectrophoretic effect: Supplementary Information

Quoc Vo[1] & Tuan Tran[1]*

[1] *School of Mechanical & Aerospace Engineering, Nanyang Technological University, 50 Nanyang Avenue, 639798, Singapore.*

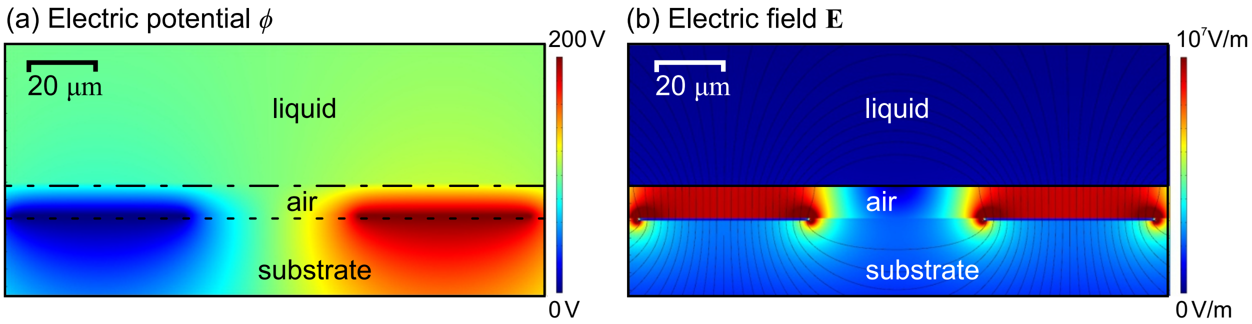

Supplementary Figure 1: Simulated electric potential and electric field generated by an interdigital electrode substrate. Simulations of (a) electric potential ϕ and (b) electric field $\mathbf{E}$ for the case that the liquid-air interface is at a distance $H = 10\,\mu\text{m}$ above the interdigital electrode substrate and the applied voltage is 200 V. The working liquid is ethanol having dielectric constant $\varepsilon_l = 24.5$. In (a): the dashed line indicates the solid surface, and the dashed-dotted line indicates the liquid-air interface. The simulation model uses the same dimensions and material properties as those of the fabricated interdigital-electrode substrate.

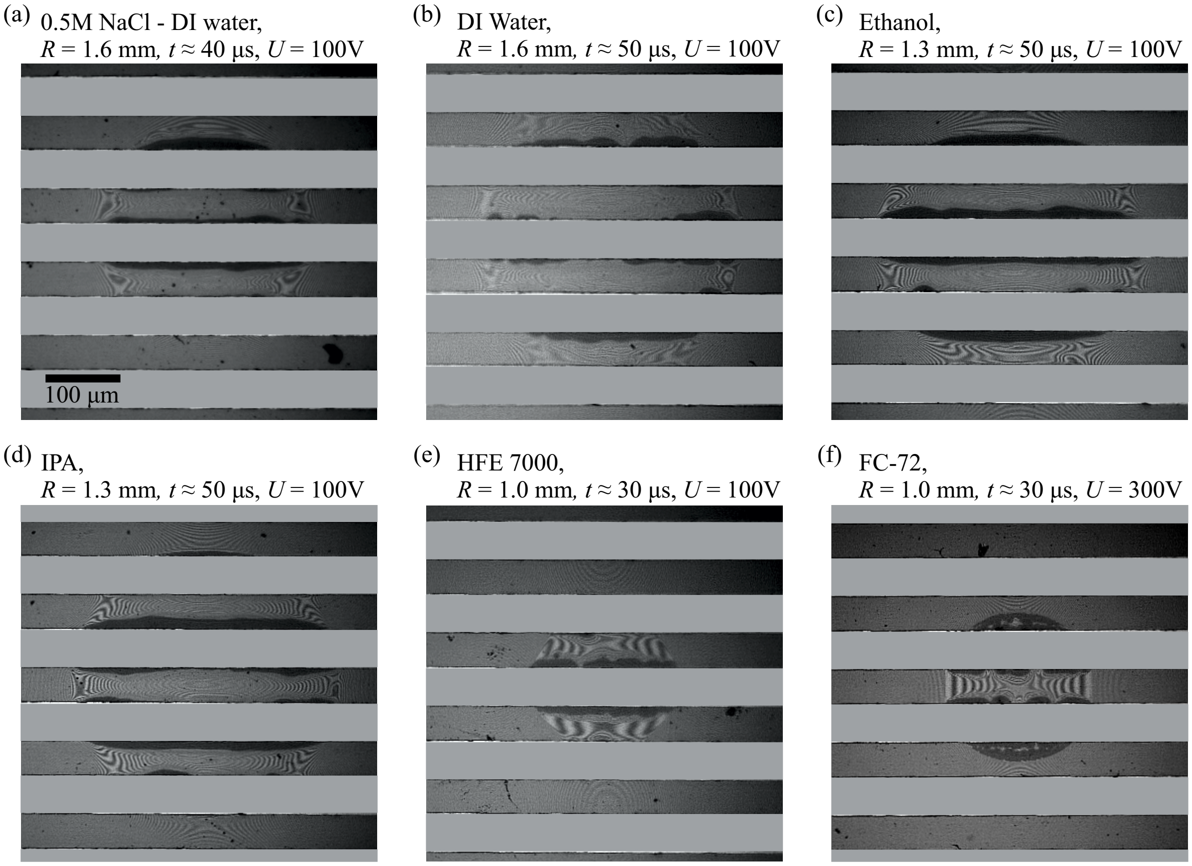

Supplementary Figure 2: Spatially periodic dielectrophoretic effect on tested liquids. Snapshots showing that the dielectrophoretic effect works on various liquids in initiating wetting at the electrode edges and forming air tunnels observable through the electrode gaps. The tested liquids include (a) Mixture of Deionized (DI) water and sodium chloride (DI water − NaCl 0.5M), (b) DI water, (c) Ethanol, (d) Iso-propanol (IPA), (e) Hydro-fluoroethers (HFE-7000, Novec), (f) Fluorinert electronic liquid (FC-72, 3M). The relevant physical properties of the tested liquids are given in Supplementary Table 1. The electrical conductivity of the DI water − NaCl 0.5M mixture is 3.02 S/m. In all experiments, the impact velocity is $v = 0.65 \pm 0.5\,\mathrm{m\,s^{-1}}$.

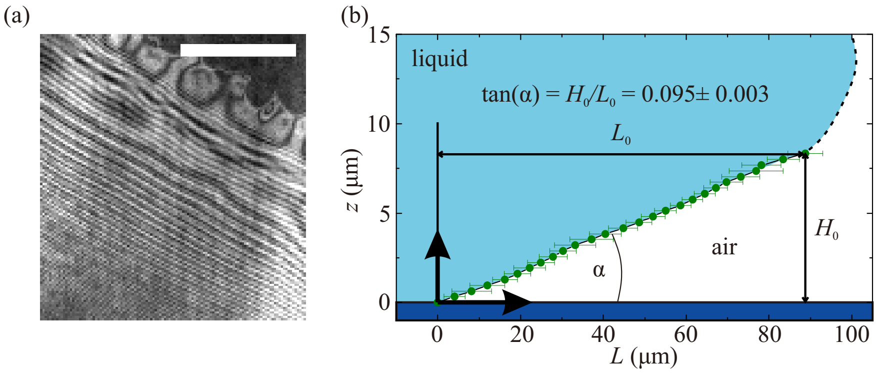

Supplementary Figure 3: Interferometric measurement of the wedge angle α. (a) Interferometric snapshot of an advancing lamella of a droplet impacting a Teflon-coated substrate with impact velocity $v = 3.1\,\mathrm{m\,s^{-1}}$. The scale bar indicates 50 μm. (b) The averaged air wedge's profile extracted along three randomly-chosen lines perpendicular to the interferometric fringe pattern.

Liquid	ε_1	$\gamma(\mathrm{mN.m^{-1}})$	$\rho\ (\mathrm{g.ml^{-1}})$
Deionized water - 0.5M NaCl mixture	N/A	80	1.00
Deionized water (DI water)	80	72	1.00
Ethanol	24.5	22.4	0.79
Isopropanol (IPA)	19.9	22	0.79
Hydro-fluoroethers (HFE 7000, Novec)	7.4	12.4	0.55
Fluorinert electronic liquid (FC-72, 3M)	1.75	10	1.68

Supplementary Table 1: Liquids used in our experiment and their properties.

Supplementary Note 1: Calculation of dielectrophoretic stress at the liquid-air interface

Electrical potential and electric field inside the liquid. In this calculation, we consider a system containing a semi-infini liquid body separated from an interdigital electrode substrate by a layer of dielectric coating and a thin air film, as shown in Supplementary Fig. 4a. The dielectric coating layer has dielectric constant ε_{c} and thickness d_{c}. The air has dielectric constant ε_{a} and thickness H. The liquid body has dielectric constant ε_{l} and extends from $z = H + d_{\mathrm{c}}$ to infinity. The electrodes's potentials are $-U/2$ and $U/2$ alternatively.

The electrical potential $\phi_{\mathrm{l}}(x, z)$ inside the liquid is calculated using the Fourier form of the solution of the Poisson's equation as [1]

$$\phi_{\mathrm{l}}(x, z) = \sum_{n=0}^{\infty} A_{\mathrm{n}} \cos\left[\frac{(2n+1)\pi}{2\delta} x\right] \exp\left[-\frac{(2n+1)\pi}{2\delta} z\right], \tag{1}$$

where $n = 0,\ 1,\ 2\ldots$ and A_{n} is the coefficient of the $n-th$ order component, and δ is the interspacing distance between electrodes. A simplified solution can be formulated by taking the first-order

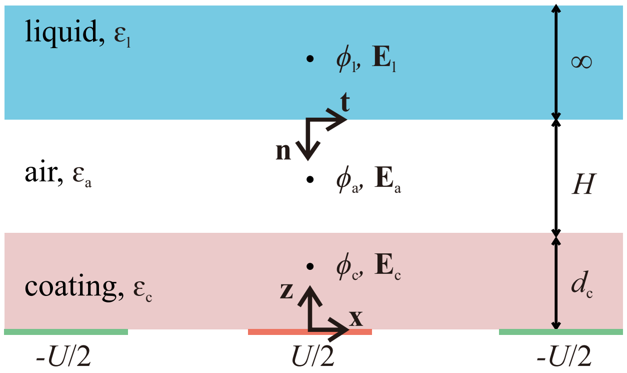

Supplementary Figure 4: Schematic (not to scale) of the liquid body above an interdigital electrode substrate.

approximation of the Fourier series:

$$\phi_{\mathrm{l}}(x,z) \approx A_0 \cos\left(\frac{x}{d_{\mathrm{p}}}\right) \exp\left(-\frac{z}{d_{\mathrm{p}}}\right), \tag{2}$$

where $d_{\mathrm{p}} = 2\delta/\pi$ is the penetration depth of the field.

By using method of images [2] to replace the dielectrics with image potentials having the same penetration depth d_{p}, we calculate the coefficient A_0 of the potential ϕ_{l} as

$$A_0 = \frac{2\varepsilon_{\mathrm{c}}}{g} \frac{2\varepsilon_{\mathrm{a}}}{\varepsilon_{\mathrm{a}} + \varepsilon_{\mathrm{l}}} \frac{8U}{\sqrt{2}\pi^2}, \tag{3}$$

where

$$g = \varepsilon_{\mathrm{c}}(1+a)\left(b\frac{\varepsilon_{\mathrm{a}} - \varepsilon_{\mathrm{l}}}{\varepsilon_{\mathrm{a}} + \varepsilon_{\mathrm{l}}} + 1\right) - \varepsilon_{\mathrm{a}}(1-a)\left(b\frac{\varepsilon_{\mathrm{a}} - \varepsilon_{\mathrm{l}}}{\varepsilon_{\mathrm{a}} + \varepsilon_{\mathrm{l}}} - 1\right), \tag{4}$$

and

$$a = \exp\left(-\frac{2d_{\mathrm{c}}}{d_{\mathrm{p}}}\right), \tag{5}$$

$$b = \exp\left(-\frac{2H}{d_{\mathrm{p}}}\right). \tag{6}$$

In our system, $d_{\mathrm{c}} = 0.5\ \mu\mathrm{m}$ and $d_{\mathrm{p}} = 32\ \mu\mathrm{m}$, allowing us to approximate $a \approx 1$ and simplify the expression for ϕ_{l}:

$$\phi_{\mathrm{l}} \approx \frac{\varepsilon_{\mathrm{r}}}{\varepsilon_{\mathrm{l}}} \frac{8U}{\sqrt{2}\pi^2} \cos\left(\frac{x}{d_{\mathrm{p}}}\right) \exp\left(-\frac{z}{d_{\mathrm{p}}}\right), \text{ for } z \geq H, \tag{7}$$

where

$$\varepsilon_{\mathrm{r}} = \frac{2\varepsilon_{\mathrm{a}}\varepsilon_{\mathrm{l}}}{\varepsilon_{\mathrm{a}}(b+1) + \varepsilon_{\mathrm{l}}(1-b)}. \tag{8}$$

The electric field inside the liquid therefore is calculated as $\mathbf{E}_\mathrm{l} = \nabla\phi_\mathrm{l}(x, z)$, or

$$\mathbf{E}_\mathrm{l} \approx \begin{bmatrix} \frac{\partial\phi_\mathrm{l}}{\partial x} \\ \\ \frac{\partial\phi_\mathrm{l}}{\partial z} \end{bmatrix} \cdot \begin{bmatrix} \hat{\mathbf{x}} \\ \\ \hat{\mathbf{z}} \end{bmatrix} = \begin{bmatrix} -\frac{\varepsilon_\mathrm{r}}{\varepsilon_\mathrm{l}} \frac{4\sqrt{2}U}{\pi^2 d_\mathrm{p}} \sin\left(\frac{x}{d_\mathrm{p}}\right) \exp\left(-\frac{z}{d_\mathrm{p}}\right) \\ \\ -\frac{\varepsilon_\mathrm{r}}{\varepsilon_\mathrm{l}} \frac{4\sqrt{2}U}{\pi^2 d_\mathrm{p}} \cos\left(\frac{x}{d_\mathrm{p}}\right) \exp\left(-\frac{z}{d_\mathrm{p}}\right) \end{bmatrix} \cdot \begin{bmatrix} \hat{\mathbf{x}} \\ \\ \hat{\mathbf{z}} \end{bmatrix}, \tag{9}$$

where $\hat{\mathbf{x}}$ and $\hat{\mathbf{z}}$ are the unit vectors in the $x-$direction and $z-$direction, respectively.

Dielectrophoretic stress at the liquid-air interface. For a medium of dielectric constant ε in an electric field $\mathbf{E}$, the Maxwell stress tensor is [3,4]

$$\mathbf{T}_\mathrm{ij} = \varepsilon_0\varepsilon \left(\mathbf{E}_\mathrm{i}\mathbf{E}_\mathrm{j} - \frac{1}{2}\delta_\mathrm{ij}\mathbf{E}^2\right). \tag{10}$$

Here, δ_ij is the Kronecker delta function. As a result, the dielectrophoretic stress at a point on the liquid-air interface having normal vector $\hat{\mathbf{n}}$ pointing from liquid to air (see Supplementary Fig. 4) is

$$\boldsymbol{\sigma}_\mathrm{e} = (\mathbf{T}_\mathrm{a} - \mathbf{T}_\mathrm{l}) \cdot \hat{\mathbf{n}}, \tag{11}$$

where $\mathbf{T}_\mathrm{a}$ and $\mathbf{T}_\mathrm{l}$ respectively are the Maxwell stress tensors in the air and the liquid at the vicinity of the liquid-air interface. We note that the electric fields $\mathbf{E}_\mathrm{l}$ in the liquid and $\mathbf{E}_\mathrm{a}$ in the air must satisfy the following boundary conditions at the liquid-air interface:

$$\mathbf{E}_\mathrm{a} \cdot \hat{\mathbf{t}} = \mathbf{E}_\mathrm{l} \cdot \hat{\mathbf{t}}, \tag{12}$$

$$\varepsilon_\mathrm{a}\mathbf{E}_\mathrm{a} \cdot \hat{\mathbf{n}} = \varepsilon_\mathrm{l}\mathbf{E}_\mathrm{l} \cdot \hat{\mathbf{n}}. \tag{13}$$

As a result, we obtain the expression for the dielectrophoretic stress at the liquid-air interface:

$$\boldsymbol{\sigma}_\mathrm{e} = \frac{1}{2}\varepsilon_0 \left[E_\mathrm{l,t}^2(\varepsilon_\mathrm{l} - \varepsilon_\mathrm{a}) + E_\mathrm{l,n}^2\varepsilon_\mathrm{l}\left(\frac{\varepsilon_\mathrm{l}}{\varepsilon_\mathrm{a}} - 1\right)\right] \hat{\mathbf{n}}. \tag{14}$$

Here, $\mathbf{E}_\mathrm{l,t}$ and $\mathbf{E}_\mathrm{l,n}$ are respectively the electric field components in the liquid along the tangential and normal directions of the interface.

Discussion on dielectrophoretic stress for conductive liquids: In this case, $\varepsilon_{\mathrm{l}} = \infty$, the electric field vanishes inside the liquid bulk. To evaluate the stress on the liquid-air interface, we apply the boundary conditions (i) $E_{\mathrm{l,\,t}} = E_{\mathrm{a,\,t}} = 0$, i.e., zero tangential electric field at the surface of a conductor, and (ii) $E_{\mathrm{l,\,n}} = E_{\mathrm{a,\,n}}(\varepsilon_{\mathrm{a}}/\varepsilon_{\mathrm{l}})$ to Supplementary Eq. 14. The resulting stress $\boldsymbol{\sigma}_{\mathrm{e}}$ becomes

$$\boldsymbol{\sigma}_{\mathrm{e}} \approx \frac{1}{2}\varepsilon_0\varepsilon_{\mathrm{a}}E_{\mathrm{a,n}}^2\,\hat{\mathbf{n}}. \tag{15}$$

Supplementary Eq. 15 indicates that the dielectrophoretic stress at the liquid-air interface is non-zero even if the liquid is perfectly conductive. Indeed, we have conducted additional experiment to confirm this effect using a conductive liquid. The result is shown in Supplementary Fig. 2a.

Supplementary Note 2: Estimation of Laplace pressure of a deformed interface

Consider a liquid body with its lower surface at a distance H away from an interdigital electrode substrate. as the dielectrophoretic force acting on the liquid surface maximises directly above the electrodes's edges, the liquid surface first deforms there. Assuming maximum deformation of the liquid surface, i.e., when the liquid surface is about to touch the substrate at the electrodes's edges, the cross-sectional area of the deformed liquid surface takes the shape of a circle with height H and base radius $\delta/2$. This results in the maximum curvature $(\delta^2+4H^2)/(8H)$ and subsequently Laplace pressure $p_{\mathrm{L}} \sim 8\gamma H/(\delta^2 + 4H^2)$, where γ is the surface tension of the liquid.

Our calculations using the relevant material properties indicates that p_{L} is significantly smaller than the dielectrophoretic stress σ_{e} for small H, i.e., well below the penetration depth $d_{\mathrm{p}} = 32\ \mu\mathrm{m}$ of the electric field. For instance, for an ethanol surface at $H = 2\ \mu\mathrm{m}$ and $U = 200\,\mathrm{V}$, the maxi-

mum dielectrophoretic stress σ_d ($\approx 5114\,\mathrm{Pa}$) is more than one order of magnitude larger than the Laplace pressure p_L ($\approx 140\,\mathrm{Pa}$). As a result, we neglect the Laplace pressure when comparing the dielectrophoretic stress and the lubrication pressure of the air flows.

Supplementary References

1. Morgan, H., Izquierdo, A. G., Bakewell, D., Green, N. G., & Ramos, A. The dielectrophoretic and travelling wave forces generated by interdigitated electrode arrays: analytical solution using Fourier series. *J. Phys. D: Appl. Phys.* **34**, 1553 (2001).

2. Griffiths, D. J., *Introduction to Electrodynamics*, 4th edition , p.121, (Pearson, London, 2013).

3. Jones, T.B. Liquid dielectrophoresis on the microscale. *J. Electrostatics* **51-52**, 290-299 (2001).

4. Mugele, F. & Baret, J.-C. Electrowetting: from basics to applications. *J. Phys.: Condens. Matter* **17**, R705–R774 (2005).

5. Brown, C. V., Wells, G. G., Newton, M. I., & McHale, G. Voltage-programmable liquid optical interface. *Nat. Photonics* **3**, 403-405 (2009).

6. Riboux, G. & Gordillo, J. M. Experiments of drops impacting a smooth solid surface: A model of the critical impact speed for drop splashing. *Phys. Rev. Lett.* **113**, 024507 (2014).
