# Supplementary material for: Mediation of lubricated air films using spatially periodic dielectrophoretic effect
Source: Nat Commun. 2021 Jul 13;12:4289. doi: 10.1038/s41467-021-24534-6 (PMC8277893; doi:10.1038/s41467-021-24534-6)
Supplement: Supplementary file 4 — Description of Additional Supplementary Files [file 41467_2021_24534_MOESM4_ESM.docx]

Description of Additional Supplementary Files

Title: Supplementary Movie 1

Description: Bottom-view recordings showing the dielectrophoretic effect in forming air tunnels and reducing air entrapment. All droplets have the same radius R = 1.3 mm and impact velocity v = 0.36 m/s.

Title: Supplementary Movie 2

Description: Splash suppression using spatially periodic dielectrophoretic force. The impact velocity is v = 3.2 m/s.
